# Supplementary material for: Are there differences between COVID-19 and non-COVID-19 inpatient pressure injuries? Experiences in Internal Medicine Units
Source: PLoS One. 2022 Feb 17;17(2):e0263900. doi: 10.1371/journal.pone.0263900 (PMC8853574; doi:10.1371/journal.pone.0263900)
Supplement: S1 File — (PDF) [file pone.0263900.s001.pdf]

COMPLEJO ASISTENCIAL UNIVERSITARIO D SALAMANCA
Paseo de San Vicente, 58-182
37007 Salamanca
Comité Ético de Investigación con Medicamentos
Teléfono: 923 29 11 00 – Ext. 55 515

Sacyl

E-mail: comite.etico.husa@saludcastillayleon.es

**EL COMITE DE ETICA DE LA INVESTIGACION CON MEDICAMENTOS DEL AREA DE SALUD DE SALAMANCA,**

**I N F O R M A**

Que el Proyecto de Investigación presentado por Dña ADELA CARPIO PÉREZ

Titulado:

**ÚLCERAS POR PRESIÓN: UNA EPIDEMIA PREVENIBLE EN EL CONTEXTO DE UNA PRÁCTICA ASISTENCIAL SEGURA**

Que presenta como Investigador/a responsable, SE AJUSTA A LAS NORMAS ÉTICAS Y DE BUENA PRÁCTICA CLÍNICA, establecidas para tales estudios.

Código CEIm: PI **2019 03 208**

Y para que conste, lo firma en Salamanca con fecha 25 de marzo de 2019

COMPLEJO UNIVERSITARIO DE SALAMANCA
COMITÉ DE ÉTICA DE LA INVESTIGACIÓN CON MEDICAMENTOS

LA SECRETARIA TÉCNICA

Fdo.: Dra. D.ª María Belén Vidriales Vicente
