# Supplementary material for: Are there differences between COVID-19 and non-COVID-19 inpatient pressure injuries? Experiences in Internal Medicine Units
Source: PLoS One. 2022 Feb 17;17(2):e0263900. doi: 10.1371/journal.pone.0263900 (PMC8853574; doi:10.1371/journal.pone.0263900)
Supplement: Attachment — Submitted filename: Response_Reviewer.docx [file pone.0263900.s005.docx]

**PONE-D-21-24374:** *Are there differences between COVID-19 and non-COVID-19 inpatient pressure injuries? Experiences in Internal Medicine Units*

**Response to Reviewers**

**Thank you to reviewer #2 for her recommendations that will increase the quality and interest of the manuscript; as well as, thank you to the editorial staff for the opportunity to send a revised version of the manuscript.**

**Reviewers' comments:**

**Reviewer #2:**

I appreciate your response to my earlier points and feel that the data presented is now much clearer. However, some of the conclusions don't seem to fit that new data. First, rates of HAPIs were low compared to a year earlier and compared to other literature, which is surprising especially as the age of patients was high with multiple comorbidities. You comment on this is "Although there is a lack of records of specific characteristics of PIs, authors considered that the assessment and management of wounds, as well as decision making of the treatment was probably carried out in situ during the assistance practice and the main reason of registered data loss were care overload and restructuring of nursing staff" which I don't really understand what you are trying to say. Are you saying that numbers of PIs were down because they weren't being accurately recorded? I think this is a major finding and requires more discussion of why you think it happened.

**RESPONSE: We have clarified this possible confusing point to improve the understanding of the discussion. As we pointed out in the discussion, the prevalence rate is lower than records of the previous year or other national studies, which may sound contradictory after taking into account the serious clinical status of the patients, the high mean age of the sample and pluripathologic condition. Regarding this point, we consider as a likely explanation that overloaded care and overall public health situation may have led to an under-registration of PIs because other registries and/or care were prioritized. Furthermore, in the discussion we tried to highlight that registered LPPs presented an incomplete record of specific characteristics of wounds such as depth, size, wound bed, etc. that were logged in some episodes. Only variables such as staging and location were completed in all episodes**

You also place emphasis in the discussion on risk-factors for PI that had a P-value of <0.05 but your analysis has a large amount of comparisons so that value isn't appropriate and needs correction for multiple comparison (eg Bonferroni or Benjamini-Hochberg) or the conclusions should be much more circumspect and this limitation strongly emphasized.

**RESPONSE: Most of the variables analysed are categorical; only some variables, for example age or hospital stay, are continuous variables that allow comparisons by pairs of group means (Bonferroni Test), as we have done. The p-values shown in the tables correspond to the bivariate analysis, as indicated in the methodological section "statistical analysis". Multivariate analysis (logistic regression) was not included in the results because it did not show statistically and clinically significant results.**

You conclude that "The study shows that HAPIs were more frequent in COVID-19 group during the first wave of COVID-19." but this is only comparing absolute numbers of HAPIs in COVID versus non-COVID. I think you need to compare rates (HAPIs per COVID patient-day versus HAPIs per non-COVID patient-day or HAPIs per COVID admission versus HAPIs versus non-COVID admission) to truly make this conclusion.

**RESPONSE: GACELA-Care® was used for data collection. It uses the *"episode of care"* as the recording unit. Only total ulcer episode data were available, as figure 1 shows; these data have been exploited in the analysis for the estimation of prevalence and incidence (Results: section 1).**

**Authors agree with the reviewers’ recommendations. We sent the manuscript as instructed.**
